# The supportive care needs survey short form 34 (SCNS-SF34): translation and cultural adaptation into the Nepali language among patients with cervical cancer in Nepal

**DOI:** 10.1186/s12955-023-02147-5

**Published:** 2023-08-23

**Authors:** Kamala Dhakal, Changying Chen, Panpan Wang, Joanes Faustine Mboineki, Mikiyas Amare Getu, Allison Boyes, Chandrakala Sharma, Bijesh Raj Ghimire, Abish Adhikari, Bibhav Adhikari, Daya Laxmi Shrestha

**Affiliations:** 1 Nursing department, The first affiliated hospital of Zhengzhou University, Jianshe Dong Lu, Zhengzhou, 450000 China; 2 School of Nursing and Health, Zhengzhou University, Zhengzhou, Henan Province China; 3 Tribhuvan University, Maharajgunj Nursing Campus, Maharajgunj, Kathmandu Nepal; 4 Institute for Hospital Management of Hanan, Longhuzhonghuan Lu, Zhengzhou, Henan 450000 China; 5 School of Nursing and Public Health, University of Dodoma, Dodoma, Tanzania; 6 The University of Newcastle (UON) University Drive, Callaghan, NSW 2308 Australia; 7 Nepal Cancer Hospital & Research Center, Hattiban, Lalitpur Nepal; 8 Kathmandu Cancer Center, Tathali, Nala Road, Bhaktapur, Nepal; 9 Little Angels’ College of Management, Hattiban, Lalitpur Nepal; 10 School of Nursing, Kathmandu University, Dhulikhel, Kathmandu Nepal

**Keywords:** Cervical cancer, Supportive care, Need assessment, Translation, Cultural validation

## Abstract

**Background:**

A questionnaire developed in one language must be translated and adapted when it will be used with patients speaking a different language and care should be taken to maintain equivalence between the source language (SL) version and its translated version. The objective of this study was to test the linguistic and cultural validity of a Nepali language version of the Supportive Care Need Survey – Short Form 34 (SCNS-SF34) used with the Nepali population.

**Methods:**

Translation of the SCNS-SF34 was carried out by following Beaton’s guidelines and Consensus-based Standards for the Selection of Health Status Measurement Instruments (COSMIN) by a research team. The translated version was administered to patients with cervical cancer in Nepal. The following steps were performed as part of the study: translation, content validity assessment, reliability assessment and measurement of errors.

**Results:**

The study reports item content validity (I-CVI) was > 0.78 and scale content validity (S-CVI) was − 0.89, 0.91 and 0.90 respectively in semantic, cultural, and conceptual aspects. The study found a content validity ratio (CVR) of 0.9 to 1, Cronbach’s α of 0.90, correlation significant at the 0.01 level (2-tailed), and clarity of the questionnaire at 91.29%. The standard error of measurement (SEM) and small detectable changes (SDC) for overall care need scores were measured 2.70 and 7.47 respectively. All items were accepted as per the original SCNS-SF34. Following the respondents’ suggestions, simpler Nepali words were chosen in some items to replace the words in the preliminary Nepali version of SCNS-SF34.

**Conclusion:**

Preliminary findings show that the Nepali translation of SCNS-SF34 is practical and applicable to the Nepali population. Financial supportive care needs, supportive care for caretakers and problems during patient hospital stays are essential to include in the questionnaire to further explore supportive care needs.

## Background

Translation of a questionnaire developed in one language becomes essential when it is being used with a population from a different language community. The direct translation of a questionnaire might distort the original meaning leading to misinterpretation. While using a questionnaire with a population that differs from the population targeted by the original version, it must be initially translated and then tested using scientific measures before being adopted so as to ensure the validity and cultural appropriateness of the translated instrument.[1, 2]. Using a previously developed and validated questionnaire helps to save time and energy and facilitates building cross-cultural knowledge. The use of the same questionnaire in different countries with similar populations helps to unite the operational definition of the constructs to be studied. This also supports multi-cultural responses for comparisons of results. However, a researcher who uses the already-developed questionnaire with a linguistically and culturally different population, should translate the original version compulsorily before being used. So, translation becomes critical step. Any deviation in translation may twist the fundamental meaning of one-to-many parts of the questionnaire. This affects the validity and reliability of the study. Moreover, the result of the study is significantly affected [2]. Therefore psychological language evaluation is necessary in order, to determine the validity and reliability of the questionnaire [3, 4]. The translated questionnaire must reflect both item and scale-level content validity, correlations, internal consistency, reliability, construct validity and responsiveness [5, 6]. Identification of the reliability and validity of the translated questionnaire can be done through a pretesting process [1]. The SCNS-SF34, which is the main focus of this paper, has been translated from United States English [7] into several languages and dialects, including Chinese [8], German [9], Turkey [3], Australian[10], Italian [11], and Dutch [12].

The SCNS & Cancer Patient Needs Questionnaire (CPNQ) are commonly used scale for the identification of supportive care needs (SCNs) [13]. The SCNS, a robust and effective cancer-specific requirement evaluation tool, assists in identifying the types and degrees of cancer patients’ requirements in five areas: psychological, health system information, sexuality, physical daily living and patient care assistance [14]. Because it was created and validated with a wide population of cancer patients based on cancer type, disease stage and period since diagnosis, the SCNS is appropriate for all types of cancer patients [15]. The SCNS is available in three different formats: 59-item long-form (SCNS-LF59) [14], 34-item short-form (SCNS-SF34) [7] and 9-item screening tool (SCNS-ST9) [16].

All versions cover the same five domains, but the SCNS-SF34 can reduce respondent burden in routine cancer care. The SCNS-LF59 and the SCNS-SF34 survey questionnaires help to identify the care needs of cancer patients [7]. The SCNS-SF34 has been found to be a reliable and accurate tool for detecting supportive healthcare needs among the cancer population in China [8], Germany [9], Australia [10], Italy [11] and Dutch [12]. It consists of 34 items along with 5 domains that include psychological needs (10 items), healthcare system and information needs (11 items), physical and daily living needs (5 items), patient care and support needs (5 items) and sexuality needs (3 items). Patients report the current need and extent of support in the previous month as a result of having cancer (1 - no need, not applicable; 2 - no need, satisfied; 3 - low need; 4 - moderate need; 5 - high need). A high tool value signifies a strong necessity for supportive care while the low tool value signifies no necessity for supportive care [7].

Regarding supportive care needs, patients with cervical cancer (CC) suffer from many physical, psychological, social, and spiritual needs that include misery, weariness, irritation, memory loss, low energy and persistent pain. Supportive care (SC) is key to improving the quality of life (QOL) of patients with CC to the highest possible level [17]. Patients with CC are reported to suffer from emotional distress and lower QOL when compared to other patients with gynecological cancers [18]. SC is required for the comprehensive and unified healthcare management of cancer patients [19]. In all stages of the disease, supportive care enhances the patients’ and their families’ ability to cope with their condition [20].

CC is ranked the fourth most predominant disease in women and the seventh most common cancer in the world. In 2018, the World Health Organization identified 570,000 new CC cases and 311,000 women who died from cervical cancer. It covers 7.5% of the total population [21]. CC is the most frequent kind of gynecological cancer in developing nations [22].

Nepal is a developing and low-income country where CC is the second most common cancer and the first most frequent cancer among women. Every year 1,928 women die out of a total of 2,942 women who are diagnosed with cervical cancer in Nepal [23, 24].

To date, the Nepali language version of SCNS-SF34 isn’t available for the assessment of SCNs. To study the SCNs of cervical cancer patients in Nepal, the researchers translated the SCNS-SF34 instrument into Nepali language. The following section describes the process of translating the original English version of SCNS-SF34 into the Nepali language along with the processes followed to check content validity, construct validity, reliability, internal consistency and clarity of the translated questionnaire.

## Methods, materials and procedure

**Fig. 1:**
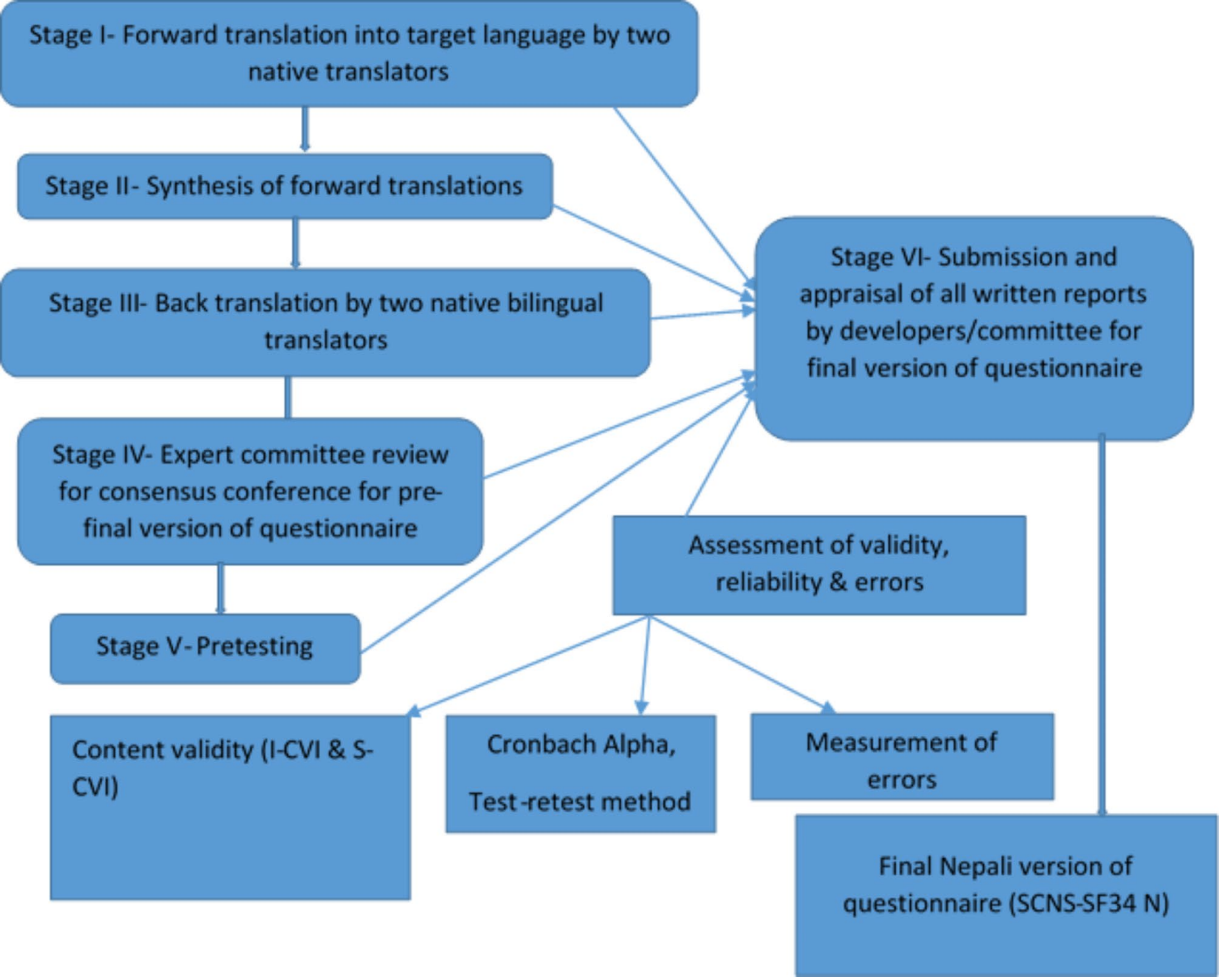
Flow diagram of translation and cultural adaptation process of SCNS-SF34 into Nepali language

The original English version of SCNS-SF34 and different versions of its Nepali translation [7] were used as the materials for this study. The translation and cultural adaptation process was carried out by following Beaton’s guidelines [1] and Consensus-based Standards for the Selection of Health Status Measurement Instruments (COSMIN)[25]. (Refer to Fig. 1)

The process comprises the translation process [1, 25], content validity assessment, clarity assessment and pretesting for cultural adaptation, reliability assessment (internal consistency, test-retest reliability) and measurement of error [25] .

Initial Nepali drafts (interim versions) were produced from which the final Nepali version was prepared. The final Nepali version underwent pretest [1], content validity [26] with clarity assessment [11], test-retest reliability [27, 28] and measurement of error [29].

### Translation process

Initial forward translation is the first step for the cultural and linguistic adaptation of a research questionnaire. The adaptation process requires at least two forward translations of the questionnaire from the source language [5]. Synthesis of forward translation is done by combining the translation of the two translators (T1, T2) and a recording observer to produce a target language draft called T12 [1]. Backward translation and synthesis are carried out from the T12 version of the questionnaire by two translators (BT1, BT2) to produce a combined original language draft called B12 [11]. The translators, without access to the original version of the questionnaire, then perform a translation of the target language, back to the original language. e A minimum of two backward translations are required to be produced by two translators [1, 5]. A consensus conference is held where experts from various fields review the translated version of the questionnaire. The expert committee approach to reviewing the translated questionnaire is essential to ensure cross-cultural correspondence in content between the source and translated versions of the questionnaire. The expert committee includes methodologists, health professionals, language professionals, and forward and backward translators [1]. Pretesting the pre-final version of the instrument with patients from the target setting is administered to 30 to 40 persons [1]. Submission and appraisal are regarded as the final stage in the adaptation process of the questionnaire. In this phase all the reports and forms are submitted to the committee for their review and approval of the final version of the translated questionnaire [1, 5].

### Cultural adaptation through content validity, clarity assessment and pretesting

In principle, experts find out the content validity index (CVI) by rating each item of the questionnaire concerning semantic/idiomatic, cultural and conceptual aspects [28, 30]. Based on the scoring using the Davis technique (1992) ,five to ten experts rate the assessment of content validity by using a 4-point Likert scale [3]. Expert opinions on content validity are taken and the CVI – is calculated in terms of item level and scale level. The content validity test is utilized via the Davis (1992) technique that grades experts’ opinions in four-choice criteria: 1 = not relevant, 2 = somewhat relevant, 3 = quite relevant, 4 = highly relevant. The CVI is calculated by dividing the number of experts that mark the choices with the total number of experts and subtracting 1. Instead of comparing this value with a statistical scale, the 0.80 value is accepted as the criterion for scale-level content validity (S-CVI) and more than 0.70 is accepted as the criterion for item level content validity index (I-CVI) [3]. For the assessment of the clarity of the questionnaire, 10–15 patients are interviewed by means of translated questionnaire using a Likert scale and comments [11]. Translated pre-final version of the questionnaire is pretested after content validity assessment to complete the cultural adaptation process [1].

### Reliability assessment (internal consistency, test-retest reliability) and measurement of error

Reliability of the translated questionnaire was established through internal consistency by performing a pretest [26] and intraclass correlation coefficient (ICC) was established by performing test-retest reliability [27]. Cronbach’s Alpha was calculated for internal consistency [28] and the ICC was calculated for test–retest evaluation [27]. A value of Cronbach’s Alpha between 0.70 and 0.90 indicates sufficiency for good internal consistency reliability [31]. A value of ICC 0.70 or above is considered a satisfactory result [32]. The median time interval between test and retest was 14 days and the ratio of the sample size to the number of items in each measure ranged from 1:1 to 1:4 [33]. According to Streiner and Norman (2003), corrected item-total correlation coefficients indicate the correlation of an item with the total scale. Cut-off values over 0.2 show a good level of correlation [34].Measurement error is the change between a measured quantity and its true value [35]. Standard error of measurement (SEM) and the smallest detectable change (SDC) are analyzed to provide clarity of the scale’s measurement ability [29]. SEM is a measure of how much measured test scores are spread around a “true” score. It is assessed from the standard deviation of a sample of scores at baseline and a test–retest reliability index of the measurement questionnaire [36]. The smaller the SEM, the more exact are the assessments that are being made. SEM was calculated by using the formula SEM = SDX ✓ (1- r), where SDX is the standard deviation of the test score and r is the reliability coefficient of the test [37]. The SDC is an amount of the variation in a scale due to measurement error [38]. It is estimated from SEM and a degree of confidence, usually 95%. Thus, a change score can only be considered to represent a real change if it is larger than the SDC. The SDC was calculated by using the formula SDC = SEM × Z, where SEM is the standard error of measurement and Z is the standard normal deviate, a value that corresponds to a specific level of confidence. Common values for Z include 1.96 for 95% [29].

### Description of the research team

The following professional members of the research team were involved in the translation of the questionnaire: a medical oncologist, a nurse working in the oncology area, a psychiatric nurse, a research nurse, a statistician, Nepali-English translators with different career specialties, a patient representative, method expert (project leader), and English and Nepali language experts [1]. The team for the content validity assessment comprised a doctor, a nurse and an educationist involved in the treatment, management, education and research of cancer patients in Nepal [26].

### Population and data collection

The data were collected from 34 patients with cervical cancer for pretest, 10 experts for the content validity assessment [1], 15 patients with cervical cancer for clarity assessment and 50 patients with cervical cancer for the test-retest method of reliability [33]. Patient samples were split among several hospitals. Pretest patients included 7 patients each from Bhaktapur Cancer Hospital (BCH); Bhaktapur; Nepal Bishweswar Prasad Koirala Memorial Cancer Hospital (BPKMCH); Bharatpur; Nepal, Nepal Cancer Hospital & Research Center (NCHRC); Harisiddhi; Nepal, and Kathmandu Cancer Center (KCC); Tathali, Nepal and 6 patients were from National Hospital & Cancer Center (NHCC); Jawalakhel; Nepal. Similarly, 3 patients from each specified hospital above were enrolled into the clarity assessment of translated questionnaire and 10 patients from each of the above specified hospitals were enrolled in the test-retest method of reliability.

### Ethical approval

#### Ethical approval

was taken from the School of Nursing and Health, Zhengzhou University, Henan, China (ZZU IRB 2019-028), Nepal Health Research Council (NHRC), Nepal (Ref. No 1706). Formal permission was taken from the participants as well as the hospitals namely BCH, Bhaktapur; BPKMCH, Bharatpur; Chitwan; NCHRC, Harisiddhi; NHCC, Jawalakhel; and KCC, Tathali for the purpose of research.

## Results - translation and adaptation process

The first is translation and cross-cultural adaptation in accordance with the Beaton’s guidelines and the second is measurement properties testing in line with the COSMIN guidelines.

### Stage (1): translation into the target language (from English into Nepali)

The independent forward translations of the original SCNS-SF34 was conducted by two Nepali-speaking English translators (T1 and T2). These English-Nepali translators, fluent in the source language and target languages, produced two Nepali versions independently and wrote comments on challenging phrases, uncertainties, and ambiguous wording in the original version. Later, poor word choices were identified and resolved in a discussion between the translators. Translation of item content, response options, and instructions was deemed equally essential. It was necessary to select forward translators from different professions to provide different perspectives and vocabularies. A translator was required to translate from the medical viewpoint with his prime focus on technical concepts communicated in and through the questionnaire. His involvement was expected to ensure content equivalency between the English version and its Nepali-translated version. The other forward translator possessed no medical knowledge and was not aware of the concepts being quantified in the questionnaire. He was expected to translate as a common language user focusing on the communication of the overall content of the questionnaire. The first forward translation was done by a medical oncologist with experience in oncology health care and clinical research. The second translation was done by a non-clinician who specialized in translation and communication. Among 34 items of the questionnaire, convergence was found in the followings items 1, 4, 5, 8, 11, 14, 15, 16, 17, 19 20, 24, 25, 26, 27, 28, 29, 30 31, 32, 33, and 34. Divergence was noted in items 2, 3, 6, 7, 9, 10, 12, 13, 18, 21, 22, and 23. (Refer to Table 1)

**Table 1:**
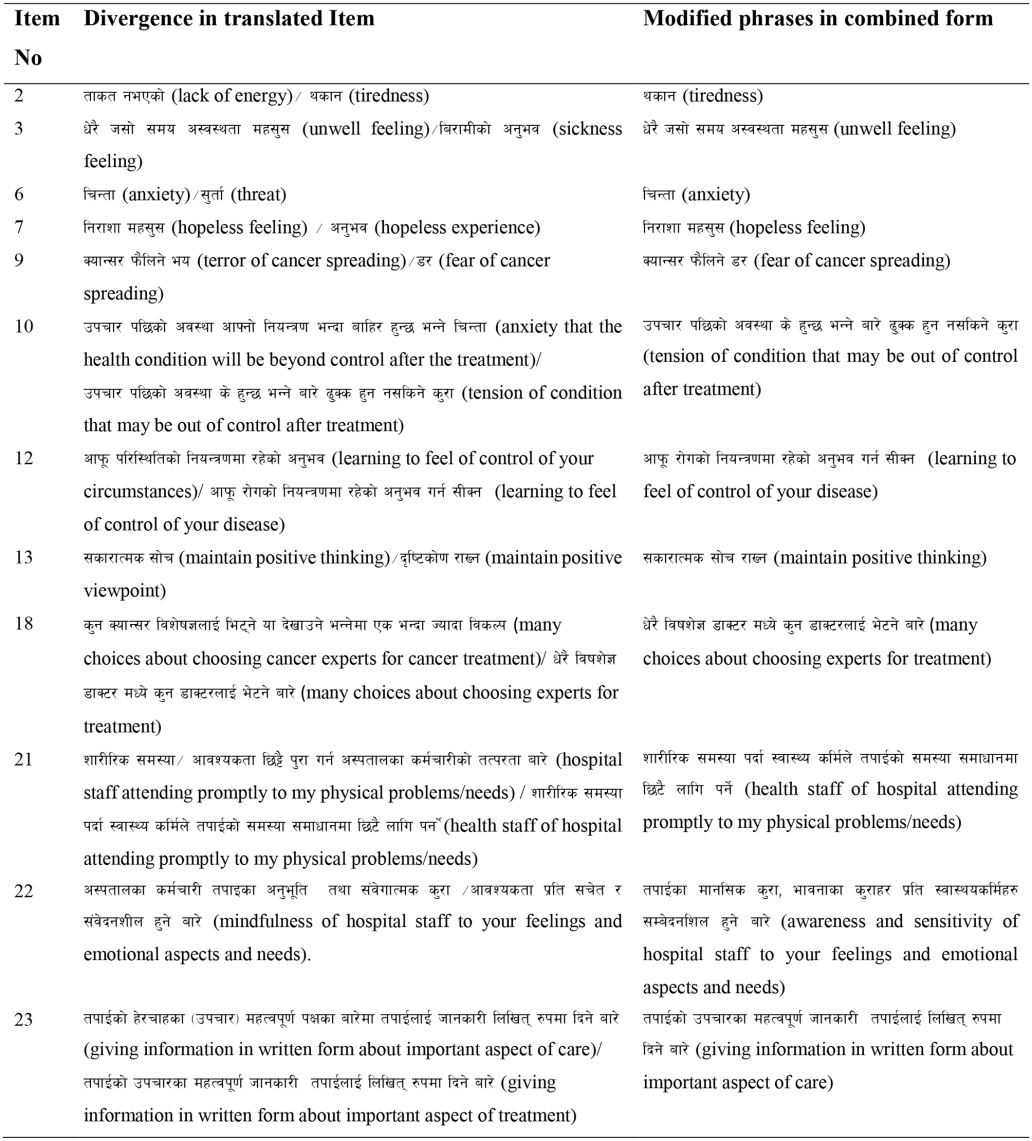
Divergence in translated items and modified phrases

### Stage (2): synthesis of forward translations (producing one document from two forward translated documents)

Two forward translated documents were merged into one by the main researcher consulting English and Nepali dictionaries. The researcher noted the main differences between the two translations and recorded them in written form. Each issue faced by both translators during forward translation was documented carefully and addressed through consensus. A Nepali language professor was consulted and simpler Nepali words were chosen as suggested to address any divergence.

### Stage (3): backward translations (from Nepali into English)

A new combined version of the forward translated document was translated back into English separately by a Nepali-English bilingual teacher and a Nepali-English translator experienced in scientific writing. These backward translators were not given the original version of SCNS-SF34. This process was carried out to ensure the validity of the translated version of the questionnaire, that is to examine whether and to what extent it reproduced the item content of the original. The comparison helped the researcher to point out the gross inconsistencies or conceptual errors in the translation. To reduce the information bias and minimize the unanticipated meanings of the items of the translated questionnaire, the two backward translators should neither have access to the original version nor should be from the medical background. The backward translated versions (BT1 and BT2) were merged by the principal investigator into one version (B12) followed by the written record of the main differences in these versions. With the help and coordination of a third Nepali-English bilingual person, item equivalence (similarity) of the synthesized backward translated version was then assessed by comparing it with the original SCNS-SF34 English version. The same words and phrases were used in 28 items by both of the backward translators, whereas synonymous words were used in six items: 10, 12, 13, 22, 30 and 32. (Refer to Table 2)

**Table 2:** The items that contained the same language reproduced by backward translators

Items No	Combined form	Original form
10	Tension that the health condition will be beyond control after the treatment	Worry that the health condition will be beyond control after the treatment
12	Feeling of being yourself under control of the situation	Learning to feel in control of your situation
13	Maintain positive thinking	Keeping a positive outlook
22	Awareness and sensitivity of hospital staff to your feelings and emotional aspects and needs	Hospital staff acknowledging, and showing sensitivity to, your feelings and emotional needs
30	Having access to professional counselling (e.g., psychologist, social worker, counsellor, nurse specialist) if you, family or friends need it	Availability of professional counselling (like psychologist, social worker, neuro specialist) to you or your family or your friends and relatives whenever needed
32	Being treated humanely and generally not only as a patient	Being treated like a person not just another case

### Stage (4): Consensus conference (preparation of preliminary Nepali version of the SCNS-SF34 questionnaire)

A consensus conference was held by a team comprising of a medical oncologist, a nurse working in the oncology area, a psychiatric nurse, a research nurse, a statistician, translators, a patient representative and the main researcher via a face-to-face meeting and email exchanges. The conference team focused its discussion on translated and synthesized documents and the key differences between the translated version and its source counterpart. The original author of the questionnaire was in touch with the expert committee throughout the process. After reviewing each translation (T1, T2, T12, BT1, BT2, and B12) and detecting divergence and convergence in meaning, the committee reached a consensus on discrepancies observed in all forms of translation through written documentation of the issues and the rationale for selecting specific words or expressions to achieve equivalence between the source and target versions. The result of the forward and backward translation was presented in this meeting. The linguistic similarities of the two synthesized translations (forward into Nepali and backward into English) were carefully analyzed by the research team and proper, simple and understandable terms were chosen for the preliminary Nepali version. After assessing equivalence in the semantic, idiomatic, experiential and conceptual areas of questionnaire, the pre-final version of the questionnaire was finalized by this expert group through a consensus conference. The consensus team continued meeting until the final format of the SCN-SF34 Nepali language assessment tool was finalized.

### Assessment of content validity and clarity of translated tool

Ten experts were consulted for the assessment of the content validity of the questionnaire. There were two oncologists, two nurses that study and work in oncology, two Ph.D. nurses, two nursing administrators of hospitals of different sizes and two professors working in research and nursing education in Nepal. I-CVI and S-CVI were assessed. I-CVI was found to be 0.78 in semantic/idiomatic, cultural and conceptual aspects and S-CVA/Ave scored 0.89, 0.91 and 0.90 in semantic/idiomatic, cultural and conceptual aspects respectively. CVR was calculated using the formula CVR = [ (E-(N/2))/(N/2)] where E indicates the number of experts who rated items as essential and N indicated the total number of experts. CVR can measure between − 1.0 and 1.0. The closer to 1.0 the CVR is, the more essential the object is. The result shows a CVR of 0.9 to 1. For the assessment of the clarity of the questionnaire, 15 patients were interviewed using the developed questionnaire that contained the Likert scale and comments. The item-wise clarity of the questionnaire and average clarity of the questionnaire were assessed. The average clarity of the questionnaire scored 91.29%. (Refer to Table 3)

**Table 3:** Content validity and clarity of the tool

SCNS-SF34	Validity (%)			Clarity%
Domains & Items	Semantic	Cultural	Conceptual	
**Physical daily living**				
1. Pain	0.93 (93)	0.95 (95)	0.92 (92.5)	95
2. Lack of energy/tiredness	0.93 (93)	0.95 (95)	0.94 (94.5)	95
3. Feeling unwell a lot of the time	0.89 (89.5)	0.92(92.5)	0.91 (91)	95
4. Work around the home	0.89 (89)	0.92 (92.5)	0.90 (90.5)	86
5. Not being able to carry on the regular tasks, which you used to do	0.87 (87.5)	0.9 (90)	0.90 (90)	91
**Total**	**0.90 (90.4)**	**0.93 (93)**	**0.91 (91.7)**	**92.4**
**Psychological**				
6. Anxiety	0.89 (89)	0.92 (92.5)	0.91 (91)	93.3
7. Feeling depressed	0.92 (92)	0.95 (95)	0.93 (93.5)	95
8. Feeling sad	0.87 (87)	0.9 (90)	0.89 (89.5)	93.3
9. Fear of spreading the cancer	0.87 (87)	0.9 (90)	0.88 (88.5)	91.6
10. The tension that health condition will be beyond control after the treatment	0.84 (84)	0.87 (87.5)	0.88 (88)	81.6
11. Uncertainty about future	0.87 (87.4)	0.9 (90)	0.89 (89)	90
12. The feeling of being yourself under control of the situation	0.84 (84)	0.87 (87.5)	0.86 (86.5)	83.3
13. Maintaining positive thinking	0.85 (85)	0.87(87.5)	0.86 (86.5)	93.3
14. Feeling the tension of death and dying	0.84 (84)	0.87(87.5)	0.86 (86)	88.3
**Total**	**0.86 (86.6)**	**0.89 (89.7)**	**0.88 (88.7)**	**0.89 (89.9)**
**Sexuality**				
15. Change in sexual experiences	0.86 (86.4)	0.90 (90)	0.89 (89.5)	90
16. Change in your sexual relation	0.91 (91)	0.94 (94.4)	0.93 (93)	91.07
31. Providing information about sexual relation	0.90 (90.5)	0.92 (92.5)	0.91 (91)	91.6
**Total**	**0.89 (89.3)**	**0.92 (92.3)**	**0.91 (91.1)**	**0.90 (90.8)**
**Patient care comfort**				
17. Worry about your loved one	0.93 (93)	0.95 (95)	0.92 (92)	96.6
18. Many alternatives about choosing doctors/experts of cancer for treatment	0.94 (94)	0.97 (97.5)	0.95 (95)	98.3
19. Many alternatives about choosing the hospital for treatment	0.91 (91)	0.95(95)	0.88 (88)	95
20. Assurance from health worker that whatever you are experiencing /thinking is normal	0.92 (92.2)	0.95(95)	0.93 (93)	91.6
21. Hospital staff attending promptly to your physical problems/needs.	0.93 (93)	0.97(97.2)	0.95 (95)	92.8
22. Awareness and sensitivity of hospital staff to your feelings and emotional aspects and needs	0.88 (88)	0.92(92.5)	0.90 (90)	88.3
**Total**	**0.91 (91.8)**	**0.95 (95.3)**	**0.92 (92.1)**	**93.7**
**Health system information**				
23. Giving information about the important aspect of your treatment and care in written form	0.87 (87.5)	0.92(92.5)	0.90 (90)	96.6
24. Giving written information, diagrams and other clear information regarding ideas to manage the disease, its symptoms and side effects at home.	0.91 (91)	0.92(92.5)	0.90 (90)	93.3
25. Sharing the treatment and investigation reports with you, which you were interested to know	0.88 (88)	0.9(90)	0.88 (88)	95
26. Giving adequate information about the treatment that you choose, its side effects before starting the treatment	0.87 (87)	0.9(90)	0.87 (87)	91.6
27. Sharing the reports of your tests/investigation with you soon as far as time allows	0.90 (90)	0.9(90)	0.88 (88)	88.3
28. Giving information about the status of cancer, its minimization or control	0.87 (87.5)	0.9(90)	0.89 (89)	90
29. Giving information about the things that you can do yourself to improve your health	0.89 (89)	0.92 (92.5)	0.89 (89)	90
30. Availability of professional counseling (like a psychologist, social worker, nurse specialist) to you or your family or your friends and relatives whenever needed	0.88 (88)	0.9(90)	0.87 (87)	86.6
32. Being treated humanely and generously not only as a patient	0.86 (86)	0.87(87.5)	0.86 (86)	86.6
33. Friendly structure/ environment in a hospital or clinic as far as possible	0.92 (92)	0.95(95)	0.93 (93)	88.3
34. Easy availability of one staff from hospital with whom you can talk about your condition, treatment and follow up	0.90 (90)	0.92(92.5)	0.91 (91)	88.3
**Total**	**0.88 (88.7)**	**0.91 (91.1)**	**0.88 (88.9)**	**90.4**
**Scale Content Validity Index (S-CVI)**	**0.89 (89.01)**	**0.91 (91.88)**	**0.90 (90.04)**	**Average Clarity 91.29**

I-CVI and S-CVI were assessed. I-CVI more than 0.7 and S-CVA more than 0.8 was accepted. Item wise clarity of questionnaire more than 80% was accepted

### Stage (5): Pretest patient survey

The translated version of the questionnaire was pretested from 1 to 2020 to 30 April 2020 to an outpatient department of the selected hospitals. It was performed to assess the clarity and understandability of the final version as well as the internal consistency of the items via Cronbach’s Alpha coefficient. Literate participants completed the self-administered questionnaire while illiterate respondents participated in face-to-face interviews with the researcher. Following the initial response, the participants were asked again about each item including their perception of the question items, difficulty level, understanding level, and cultural appropriateness of words and phrases. They were encouraged to give comments on any section of the questionnaire so that the final Nepali version would have higher content efficacy and cultural acceptability. Pretest respondents included 34 patients with cervical cancer representing the target population of the study. They were female patients > 18 years of age with any stage or treatment setting, and other socioeconomic characteristics. Among 34 respondents, most of the respondents (35.3%) were over 60 years and 29.4% of them were between 46 and 55 years. Most of the respondents (64.7%) were illiterate. 70.6% of them were married and 55.9% of them were in Stage II of cervical cancer. The modality of treatment for the majority of the respondents (67.6%) was both radiation and chemotherapy. (Refer to Table 4)

**Table 4:** Demographic characteristics of respondents (n = 34)

Variables	Frequency	Percent
**Age**		
<= 45.00	6	17.6
46.00–55.00	10	29.4
56.00–60.00	6	17.6
61.00+	12	35.3
Mean Age	55.64+_13.77	
**Education**		
Illiterate	22	64.7
Literate	12	35.2
**Marital status**		
Married	24	70.6
Single/Widow	10	29.4
**Stage of disease**		
Stage I	2	5.9
Stage II	19	55.9
Stage III	12	35.3
Stage IV	1	2.9
**Treatment modalities**		
Radiation	6	17.6
Operation + Chemotherapy	1	2.9
Operation + Radiation	2	5.9
Radiation + Chemotherapy	23	67.6
Operation + Chemotherapy + Radiation	2	5.9
**Total**	**34**	**100**

The frequency (n), percentage (%), mean and standard deviation (SD) were used for data analysis

### Reliability and measurement error of the translated tool

Reliability was assessed through internal consistency during pretest and ICC during test-retest. During pretest, 34 respondents were invited to respond on the five-point Likert scale. Scale mean, scale variance, total correlation and Cronbach’s alpha were calculated. The reliability was confirmed after evaluating the internal consistency by using Cronbach’s α coefficient. Item-wise and domain-wise Cronbach’s alpha was 0.7 and composite Cronbach’s alpha scored 0.90. The results of corrected item-total correlation of all items were higher than 0.2, indicating there is a high level of correlation within all the items of the Nepali version questionnaire of SCNS-SF34. (Refer to Table 5).

**Table 5:** Internal consistency of the tool (n = 34)

Domains and items of SCNS-SF34	Scale Mean if Item Deleted	Scale Variance if Item Deleted	Corrected Item-Total Correlation	Cronbach’s Alpha if Item Deleted
**Physical daily living**				
1. Pain	11.53	17.651	0.275	0.907
2. Lack of energy/tiredness	11.50	15.167	0.615	0.898
3. Feeling unwell a lot of the time	11.35	13.316	0.727	0.897
4. Work around the home	11.06	13.027	0.748	0.897
5. Not being able to carry on the regular tasks, which you used to	11.18	14.392	0.780	0.898
**Cronbach’s Alpha, physical daily living**				**0.899**
**Psychological**				
6. Anxiety	28.26	37.413	0.672	0.916
7. Feeling depressed	28.62	39.213	0.560	0.924
8. Feeling sad	28.32	37.013	0.757	0.910
9. Fear of spreading the cancer	28.00	37.091	0.821	0.906
10. The tension that health condition will be beyond control after the treatment	28.12	37.986	0.814	0.907
11. Uncertainty about future	28.09	37.598	0.731	0.912
12. The feeling of being yourself under control of the situation	28.03	39.242	0.737	0.912
13. Maintaining positive thinking	28.24	39.701	0.672	0.916
14. Feeling the tension of death and dying	27.97	37.484	0.760	0.910
**Cronbach’s Alpha, psychological**				**0.921**
**Sexuality**				
15. Changes in sexual experiences	5.79	9.441	0.931	0.906
16. Changes in your sexual relation	5.82	9.544	0.929	0.908
31. Providing information about sexual relation	5.50	8.924	0.845	0.976
**Cronbach’s Alpha, sexuality**				**0.951**
**Patient care support**				
17. Worry about your loved one	15.53	15.166	0.418	0.899
18. Many alternatives about choosing doctors/experts of cancer for treatment	15.76	12.367	0.531	0.897
19. Many alternatives about choosing the hospital for treatment	16.00	12.182	0.566	0.897
20. Assurance from health worker that whatever you are experiencing /thinking is normal	16.32	14.044	0.573	0.897
21. Hospital staff attending promptly to your physical problems/needs.	16.41	13.704	0.450	0.899
22. Awareness and sensitivity of hospital staff to your feelings and emotional aspects and needs	16.59	14.856	0.376	0.900
**Cronbach’s Alpha, patient care support**				**0.898**
**Health system information**				
23. Giving information about the important aspect of your treatment and care in written form	30.03	34.939	0.471	0.842
24. Giving written information, diagrams and other clear information regarding ideas to manage the disease, its symptoms and side effects at home.	30.03	31.996	0.779	0.813
25. Sharing the treatment and investigation reports with you, which you were interested to know	30.44	35.163	0.541	0.835
26. Giving adequate information about the treatment that you choose, its side effects before starting the treatment	30.56	33.890	0.641	0.826
27. Sharing the reports of your tests/investigation with you soon as far as time allows	30.68	36.225	0.436	0.844
28. Giving information about the status of cancer, its minimization or control	30.26	36.988	0.404	0.846
29. Giving information about the things that you can do yourself to improve your health	30.53	36.681	0.452	0.842
30. Availability of professional counseling (like a psychologist, social worker, nurse specialist) to you or your family or your friends and relatives whenever needed	29.91	33.840	0.604	0.830
32. Being treated humanely and generously not only as a patient	31.00	37.758	0.468	0.841
33. Friendly structure/ environment in a hospital or clinic as far as possible	30.88	38.955	0.394	0.824
34. Easy availability of one staff from hospital with whom you can talk about your condition, treatment and follow up	30.38	34.122	0.672	0.899
**Cronbach’s Alpha, health system information**				**0.848**
**Composite Cronbach’s Alpha)**				**0.902**

Reliability was evaluated using Cronbach’s Alpha coefficient. Item wise Cronbach’s alpha more than 0.7 was accepted

### Instructions and response scale

The majority of respondents said that the instructions related to the level of needs [1–5] must be included not only in the questionnaire section but also in the example section to make the instruction and response scale clearer. Half of the respondents suggested substituting the example of a perceived need in the instruction section i.e. *being informed about things you can do to help yourself to get well* in the original version with the example related to a physical need (pain) because it was easier for them to relate to the example about pain than the example given in the original version.

### Item clarity, comprehensiveness, significance and modified phrases

Almost all respondents found all items understandable in their respective contexts. The team members agreed to aid alternatives related to the level of needs [1–5] in the instruction and other sections and an example related to a physical need (pain) was modified. As suggested by the respondents, simple Nepali words and phrases were chosen in Items 2, 3, 6, 7, 9, 10, 12, 13, 18, 21, 22 and 23 in the preliminary Nepali version. (Refer to Table 6)

**Table 6:**
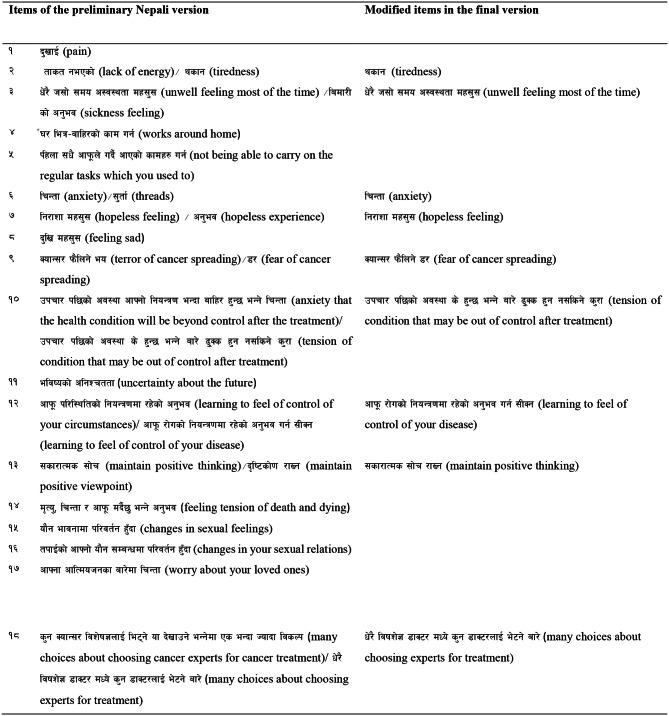

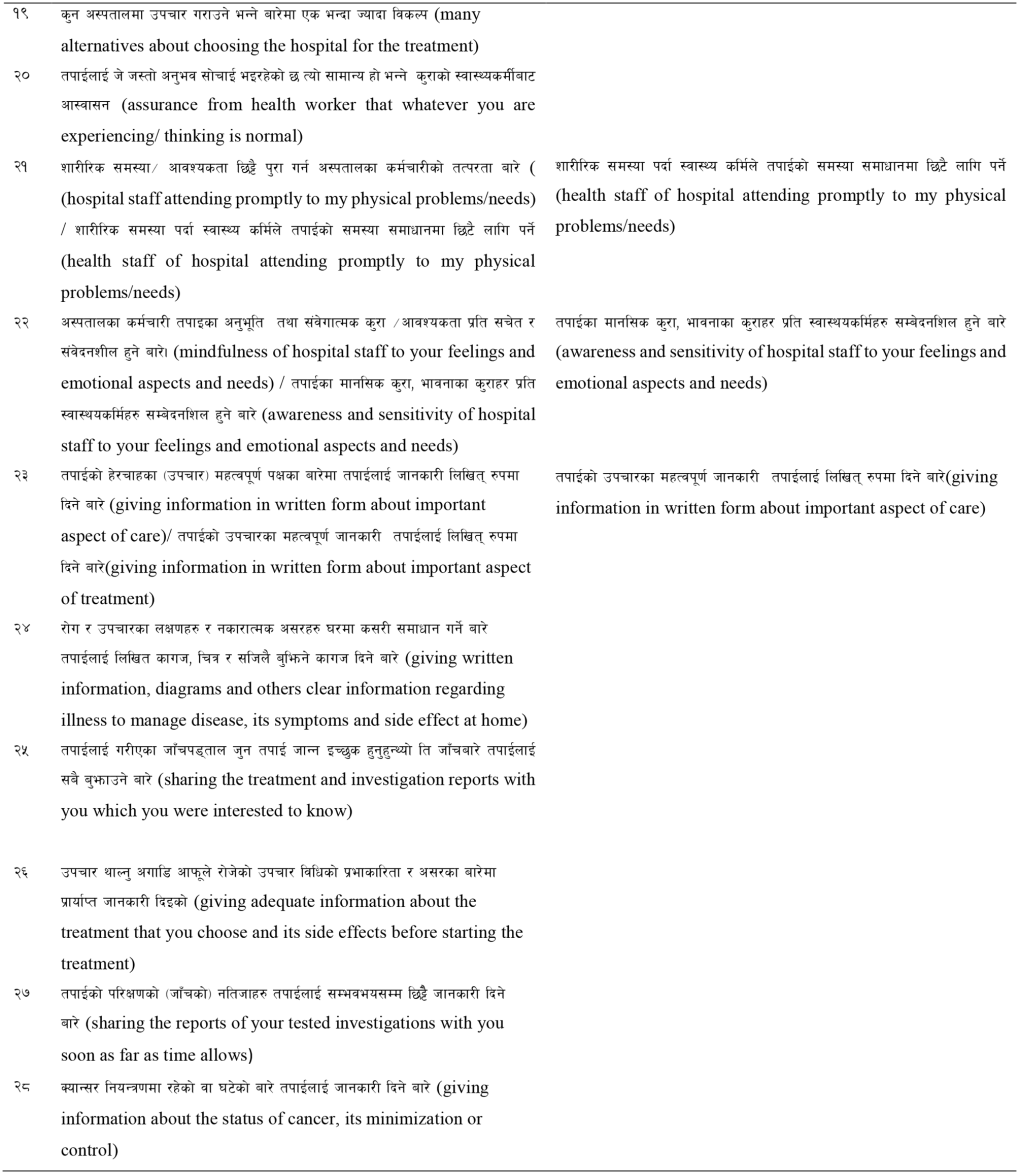

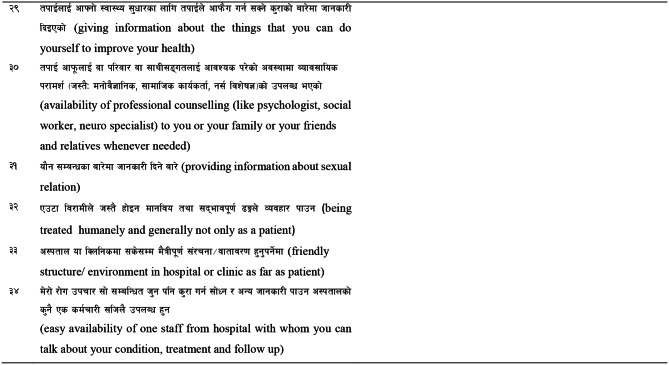
Preliminary Nepali version of SCNS-SF34 and modified items in the final version

### Item importance

Most of the study respondents graded the significance of each item as ≥ 3 on a five-point Likert scale, and 85% of them rated each one as ≥ 4.

### Item acceptability

All the study respondents accepted all the items. All of the patients commented positively on Item 14 (feelings about death and dying). Despite being a distressing concept, the inquiry about this feeling of death was deemed essential to make the patient more comfortable. All the respondents felt that Items 15 (change in sexual desires) and 16 (change in sexual relations) were embarrassing because talking about sex-related concepts is not so common among Nepali women. Despite these items needed to be included in the questionnaire to explore the patients’ sex-related problems. All the respondents suggested including in the questionnaire problems concerning finance, caretakers, and accommodations during the treatment.

### Consistency assessment through intra-class correlation coefficient, SEM and SDC

The intra-class correlation coefficient was assessed using the Pearson correlation coefficient test between the score of test and retest. The test-retest method was conducted among 50 respondents. The retest was carried out after 3 weeks of completion of the test assessment.

Table 7 shows that there is a significant correlation coefficient between each domain of SCNS-SF34 during the test and retest, as the p-value was found to be less than 0.01. This signifies that there is high consistency between test and retest scores. The SEM and SDC for health system information was found to be 3.31 and 7.74 respectively. Whereas the SEM and SDC for overall care needs were found to be 2.70 and 7.47 respectively. The results of this study indicate that the SDC value of each domain of SCNS-SF34 N were higher than SEM, so the changes in the scores represent a real change. (Refer to Table 7)

**Table 7:** Intra-class co-relation coefficient with SEM and SDC of test-retest score

Domain of SCNS-SF34	N	Test score		Re-test score		ICC (r)	SEM	SDC	p-value
		Mean	SD	Mean	SD				
Physical daily living	50	46.40	23.93	51.90	23.62	0.91	4.76	13.18	0.000
Psychological	50	60.55	22.73	64.35	23.72	0.98	4.65	12.88	0.000
Sexuality	50	38.33	35.48	39.67	35.46	0.98	5.09	14.66	0.000
Patient care support	50	48.90	20.78	47.80	20.03	0.97	4.08	11.31	0.000
Health system information	50	49.73	16.86	49.00	16.25	0.97	3.31	9.18	0.000
Overall care need score	50	48.78	13.68	50.54	13.27	0.98	2.70	7.47	0.000

ICC = intraclass correlation coefficient; significant at 0.01 level; SD = standard deviation; SEM = standard error of measurement; SDC = smallest detectable change

### Stage (6): approval from the research team and original author

In this phase, all the reports and forms were submitted to the research team and original author for their approvals to finalize the instrument in the target language. Statistical Package for Social Science (SPSS) version 20 software was used for data entry, coding and analysis by the main author to discover difficult areas of the translated version and other related parameters. The findings of an analysis of the pretest, validity, clarity, reliability, measurement error assessment and consensus conference reports of the pre-final version were discussed and distributed to the research team. Following all these procedures after getting approval of the research team, the final synthesized Nepali version of SCNS-SF34 was used in the subsequent psychometric validation study.

### Approval from original authors

All the documents related to the translation and transcultural adaptation process were sent to the original authors of the questionnaire for further approval and validation of the translated and validated questionnaire. After the approval from the original author, the Nepali version of SCNS –SF34 questionnaire was used for psychometric validation. (Refer to Table 6)

## Discussion

The translation and cross-cultural adaptation of the English version of the SCNS-SF34 into the Nepali language was followed by confirmation of the content validity, reliability, measurement of errors and confirmation of construct validity of the Nepali version of the SCNS-SF34.

The processes followed in the translation and cultural adaptation of this questionnaire for Nepali patients with cervical cancer echo the processes followed in the translation and adaptation of this tool for other types of cancer patients in Italy [11], Turkey [3], China [8] and Germany [9].

The analysis further revealed that I-CVI was more than 0.78 and the Scale level content validity index (S-CVI) was 0.91. The content validity ratio (CVR) was found to be between 0.9 and 1. A study in Turkish reported I-CVI of the SCNS-SF34 being 0.80-1.00 and the S-CVI of the scale being 0.83 [3]. The assessment of content validity is vital to confirm the full range of knowledge and aspects of the psychological constructs. It is essential to measure the adequacy with which a measure assesses the domain of interest [39].

The item-wise Cronbach’s alpha was found to be more than 0.7 and the average Cronbach’s alpha was recorded 0.902. Correlation is significant at the 0.01 level (2-tailed). The Cronbach Alpha coefficient was 0.93 [3] in the adaptation of the Short-Form Supportive Care Needs Survey Questionnaire (SCNS-SF34) in Turkish for breast cancer patients. The finding of the present study is further supported by the study “Supportive care needs and quality of life of patients with gynecological cancer undergoing therapy in Indonesia”- that used the tool SCNS-SF34 and found that it had a reliability score of 0.933 [40]. Likewise, the study “Un-met Supportive Care Needs of Iranian Breast Cancer Patients” found that internal reliability coefficient (Cronbach alpha) of the translated questionnaire was substantial, greater than 0.90 [41], which also supports the finding of the present study. Identification of Cronbach’s Alpha is essential to calculate the internal consistency of the scale items [39]. It helps to identify the degree to which the set of items in the scale co-vary relative to their sum score. It is the most common scale and seems to have received approval if it is found to be at the acceptable level. The value of the alpha coefficient of 0.70 has often been considered as a satisfactory range for reliability as cited in best practices for developing and validating scales for health, social, and behavioral research: a primer [39]. According to the result of this present study, the Cronbach’s alpha if item deleted shows is pain was deleted in physical domain increases to 0.907 from 0.899 and providing information about sexual relations in sexuality domain it increases to 0.96 from 0.951; however both are important items which are also suggested by original scale [7] and is above the Cronbach’s alpha threshold of 0.7 [31]. Sample size effects may be the reason why the value of physical daily living sub-scale Cronbach’s alpha (0.899) is lower than the value of Cronbach’s alpha if deleted (pain 0.907) and the value of sexuality sub-scale Cronbach’s Alpha (0.951) is lower than the value of Cronbach’s Alpha if item deleted (providing information about sexual relations, 0.96) [42]. If both items were deleted it also affects criterion validity [42]. The subscale Cronbach’s Alpha is also lower than the value of Cronbach’s Alpha if deleted but it remains more than the required value of Cronbach’s Alpha 0.7 [31]. Both these items are thus retained in the Nepali version of SCNS-SF34.

The average clarity of the translated questionnaire was 91.29%. A study in Italy regarding the translation of SCNS-SF34 found that all study respondents considered all items were clear and comprehensible only 15.5% of the respondents reworded the third item [11]. A clear questionnaire can be responded by the respondents easily. This helps to reduce information bias and improves the validity and reliability of the study [43].

All items of the original version of the questionnaire were accepted in the current study and the twelve items modified in the final version of the translated questionnaire were described in Table 1. A similar finding has been reported in the study performed in Italy concerning the translation of SCNS-SF34 [11]. The study reported the acceptance of all items and modification of Items 10, 21 and 22 [11]. Unlike the present study, it also modified Items 5, 17, 20, 24, 25, 26, 27, 28 and 31 [11]. In contrast to the finding of the current study, Item 14 was deleted in one study carried out in mainland China [8]. Modification, addition or removal of items can occur as per the cultural and language differences between the original version and its translation. Adaptation and modification are necessary to make the items in the questionnaire congruent with the target language and culture [3]. Unlike in the Chinese translation, Item 14, related to death and dying, was retained in the Nepalese translated version. Respondents of the current study felt no difficulty in sharing their feelings about death and dying. The majority of people in Nepal follow Hinduism and Buddhism, each religious community having its own beliefs and practices about death and dying. According to Hindu philosophy death is a process through which the soul transfers to the next life. Death and dying are seen as a natural and cyclic process leading to more support for non-aggressive end-of-life care [44]. This item was also commented on by respondents reporting the cultural and linguistic adaption of the SCNS-SF34 into Italian [11].

The results in this study signify there is high consistency between test and retest through ICC which indicates there is significant correlation between each domain of SCNS-SF34 during test and retest (p-value less than 0.05). An adaptation study in Turkey used the split half method for the assessment of ICC. Guttmann Split-Half coefficient was found to be 0.73 [3]. ICC is a measure of the reliability of two different raters to measure subjects similarly. ICC is carried out for the assessment of the reliability of measurement scales [45].

### Strengths and limitations of the study

The present study has followed standard Beaton’s guidelines for the translation and cross-cultural adaptation and COSMIN guidelines for measurement properties of the SCNS-SF34 into Nepali language.

The present study reveals some limitations. This scale has only been translated and culturally adapted in Nepali for patients with cervical cancer. Suggestions for further research include the translation and adaptation for patients with different types of cancer. Additionally, the questionnaire should be translated for other major languages used in Nepal communities. And the instrument should be validated for patients who are receiving treatment in different clinical settings beyond outpatient therapies.

## Conclusion

The present study reported the processes of translating and adapting the original English version of the SCNS-SF34 into Nepali and the validation of the translated version to elicit valid and reliable information from cervical cancer patients in Nepal. The finding shows that the preliminary Nepali version of SCNS-SF34 is relevant and effective with the Nepali population. Taken together, the respondents accepted certain questionnaire items as they are in the original version, while they suggested modification of certain items for better comprehensibility, and suggested adding finance-related supportive care needs, supportive care needs of caretakers and accommodation problems during the hospital stay.

We are further studying on the validation of the questionnaire on a larger sample of the target population in different cancer hospitals in Nepal. The measurement properties are being determined in a larger sample are factor structure and factor loadings (through exploratory factor analysis (EFA) and confirmatory factor analysis (CFA), Eigenvalues, floor effect, ceiling effect, variance, construct validity, convergent validity and discriminant validity and structural validity. In particular, the research team aims to prospectively assess the supportive care needs of cervical cancer patients along with associated factors.

## Data Availability

The primary data are stored safely and confidently by the corresponding authors and agree to review the primary data upon the request of the journal. The questionnaire can be available upon request through authors.
